# A randomized, placebo-controlled, double-blinded pilot study of angiotensin 1–7 (TXA-127) for the treatment of severe COVID-19

**DOI:** 10.1186/s13054-022-04096-9

**Published:** 2022-07-28

**Authors:** Gebhard Wagener, Monica P. Goldklang, Adam Gerber, Katerina Elisman, Katherine A. Eiseman, Laura D. Fonseca, Jeanine M. D’Armiento

**Affiliations:** 1 Department of Anesthesiology, Columbia University Irving Medical Center, 622 West 168th Street, PH 5, New York, NY 10032 USA; 2 Columbia University Vagelos College of Physicians and Surgeons, New York-Prebysterian Columbia University Medical Center, 630 West 168th Street, New York, NY 10032 USA

*Trial registration* ClinicalTrials.gov Identifier: NCT04401423.

Coronavirus disease 2019 (COVID-19) is associated with acute respiratory distress syndrome and multi-organ failure. SARS-CoV-2 enters cells through angiotensin-converting enzyme 2 (ACE-2), resulting in endocytosis and translocation. During this process, ACE-2 is phagocytosed and rendered non-functional [1]. ACE-2 mediates the conversion of angiotensin II to angiotensin 1–7, a vasodilator that opposes the effect of angiotensin [2, 3]. Reduction of ACE-2 likely causes a decrease in conversion of angiotensin I and II with excess angiotensin II causing vasoconstriction, oxidative stress, inflammation, apoptosis, fibrosis and water and solute retention. The hyperinflammatory state and acute kidney injury in COVID-19 could be in part explained by a reduction of ACE-2 and angiotensin 1–7.

TXA127 is pharmaceutically formulated angiotensin (1–7) and has been studied in murine models of lung injury [4] and several small clinical trials in humans, with only minor adverse effects [5]. We hypothesized that TXA-127 replaces physiologic levels of angiotensin 1–7 and ameliorates multi-organ failure in COVID-19 and therefore designed a single-center, double-blinded, randomized placebo-controlled pilot study of TXA-127 in patients hospitalized with severe COVID-19 and respiratory symptoms. The aim of this study was to ascertain the safety of TXA-127 in COVID-19 and obtain data for the design of larger trials. An investigator-initiated new drug application (IND) was approved by the Food and Drug Administration (FDA), and the study was approved by the Columbia University Irving Medical Center (CUIMC) Institutional Review Board (IRB #AAAT0535).

After informed consent, we included adult patients over the age of 18 with COVID-19 confirmed by polymerase chain reaction (PCR) admitted to the hospital requiring supplemental oxygen. Intubated patients or those with chronic or acute kidney disease were excluded. Participants were double-blinded randomized to TXA-127 0.5 mg/kg daily or placebo intravenously for 10 days. The primary endpoints were acute kidney injury (AKI) (increase of creatinine > 0.3 mg/dL or 50% above baseline) and respiratory failure requiring ventilatory support.

Seven hundred and twenty patients were assessed from February 09, 2021, to May 17, 2021, and after exclusion of 698 patients, 22 participants were randomized. Patients were recruited 3.8 ± 2.8 days after admission and 10.1 ± 3.5 days after onset of symptoms. Two patients, both randomized to placebo never received placebo: one patient withdrew and one required mechanical ventilation prior to placebo administration. We included 20 subjects in the analysis. At randomization, 15 patients required oxygen by nasal cannula, 4 by high flow nasal cannula and 1 using noninvasive ventilation (Table 1 demographics).

**Table 1:** Patient demographics and clinical characteristics. (n: number of participants; IQR: interquartile range; none of the comparisons were significantly different)

Demographics and Clinical Characteristics		Placebo (*n* = 9)	Drug (*n* = 11)
Age median (IQR)	Years	55 (48–66)	57 (45–61.5)
Sex n(%)	Male	4 (44.4%)	9 (81.8%)
	Female	5 (55.6%)	2 (18.2%)
Oxygen at randomization n(%)	Room air	0	0
	Nasal cannula	8 (88.9%)	7 (63.6%)
	High flow nasal cannula	1 (11.1%)	3 (27.3%)
	Noninvasive ventilation/BiPAP	0	1 (9.1%)
Days median(IQR)	Begin symptoms to admission	7 (7–8)	8 (2–9.5)
	Admission to consent	3 (2–4)	3 (2–5)
	Length of hospital stay	7 (6–8)	11 (6–21)
	Days of drug-/ placebo-administered	5 (4–6)	4 (2.5–8.5)

TXA-127 was to be administered for 10 days; however, hospitalizations were often shorter. Participants received drug/placebo for 4.5 (3–7.25) days [4 (2.5–8.5) days for drug and 5 (4–6) days for placebo, *p* = 0.736]. Only three patients received drug/placebo for 10 days (two drug, one placebo). Sixteen participants were discharged before ten days, one participant’s clinical condition deteriorated, and three withdrew from the study. The major adverse effects were cough, headache and chest discomfort, but not hypotension or fever (no difference between groups).

Four participants required mechanical ventilation (three drug, one placebo). Of these, three died: one 22 days after admission (TXA127 given for three days), one 44 days after admission (TXA127 given for ten days) and one 55 days after admission (placebo given for 10 days). There was no difference between the groups with regard to intubation, length of stay, and mortality. Four patients developed acute kidney injury based on the kidney disease improving global outcomes (KDIGO) criteria (three drug and one placebo; no difference between the groups).

This was the first described use of angiotensin (1–7)/TXA-127 in patients with severe COVID-19. No serious adverse events were observed with TXA-127, similar to studies for other indications. The three subjects who withdrew from the study did not experience adverse effects associated with drug administration. While the study was clearly underpowered to detect a difference in outcome, it provides important data to design larger studies. Much of the underpowering was related to the very high incidence of AKI and mechanical ventilation at our center in April 2020, the time of the initial study design. During study recruitment in the second wave, treatment paradigms for COVID-19 changed and affected outcomes. As a result, morbidity and mortality improved by the time patients were recruited into this study. Early termination of the study for safety analysis was elected given the imminent start of an NIH-funded multicenter study of the efficacy of TXA127 in COVID-19 infection. Only three patients completed a full course of ten days of study drug/placebo administration mostly because patients recovered quickly and were discharged. Due to decreasing admission rates and low length of stay, the trial was terminated. Future larger trials should account for differences in outcomes and consider a study design that allows adjustment of the study size if the incidence of endpoints is different than previously expected.

In conclusion, in this proof-of-concept study, TXA-127 was safe to administer in patients with severe COVID-19 infection. The combined primary endpoints of acute kidney injury and/or respiratory failure requiring mechanical ventilation happened infrequently, underpowering the analysis.

## Data Availability

The datasets used and/or analyzed during the current study are available from the corresponding author on reasonable request.

## Abbreviations

COVID-19: Coronavirus disease 2019
ACE-2: Angiotensin-converting enzyme 2
PCR: Polymerase chain reaction
